# Associations between psychological distress and the most concerning present personal problems among working-age men in Japan

**DOI:** 10.1186/s12889-015-1676-7

**Published:** 2015-03-31

**Authors:** Koji Wada, Hisashi Eguchi, Daisuke Yoneoka, Jun Okahisa, Derek R Smith

**Affiliations:** International Health Cooperation, National Center for Global Health and Medicine, 1-21-1 Toyama, Shinjuku-ku, Tokyo 162-8655 Japan; Department of Public Health, Kitasato University School of Medicine, 1-15-1 Kitasato, Minami-ku, Sagamihara, Kanagawa 252-0374 Japan; Department of Statistical Science, School of Advanced Sciences, the Graduate University for Advanced Studies, Kamiyamaguchi Midori-Cho 10-3, Tachikawa-Shi, Tokyo 190-8562 Japan; Employee Health Office, Oriental Consultants Co. Ltd, 3-12-1 Honmachi, Shibuya-ku, Tokyo, 151-0071 Japan; School of Health Sciences, Faculty of Health and Medicine, University of Newcastle, Brush Road, Ourimbah, New South Wales, 2258 Australia

**Keywords:** Bullying, Personal problems, Personal relationships, Purpose in life, Working age population

## Abstract

**Background:**

Personal problems are known to influence mental health among workers. The current study investigated the most concerning present personal problems which have the greatest impact on psychological distress among working-age Japanese men, rather than issues relating to work tasks or duties.

**Methods:**

We obtained data from the 2010 Comprehensive Survey of Living Conditions conducted by the Ministry of Health, Labour and Welfare. The original survey interviewed 289,363 households in 5,150 randomly selected municipalities of Japan, from which 228,664 households agreed to participate. We analyzed the data pertaining to men who were 20 to 59 years of age and the head of a family. The questionnaire included occupation, employment status, the most concerning present personal problems, and a measure of psychological distress (the Kessler 6 scale). Multiple logistic regression analysis was conducted to delineate the association between present personal problems and psychological distress.

**Results:**

A total of 76,950 males were included in the analysis, 27.7% of whom reported some type of psychological distress. Statistical analysis revealed that psychological distress was associated with bullying and harassment (Odds Ratio (OR): 2.05, 95% Confidence Interval (95% CI): 1.50-2.56), divorce (OR: 1.90, 95% CI: 1.53-2.26), concerns about one’s purpose in life (OR: 1.73, 95% CI: 1.59-1.88), personal relationships with family members (OR: 1.49, 95% CI: 1.37-1.60), personal relationships with others (OR: 1.38, 95% CI: 1.29-1.48), own diseases (OR: 1.24, 95% CI: 1.15-1.33), and financial difficulties (OR: 1.16, 95% CI: 1.12-1.20); when compared with problems related to work tasks or duties.

**Conclusions:**

Several personal factors appear to have a greater impact on the mental health of Japanese men of working age, when compared to the influence of work tasks or duties. Asking workers directly about the problems that most concern them in life might help better identify those in need psychological support. Multidisciplinary interventions to address their life concerns will likely be necessary for solving these problems and reducing psychological distress.

## Background

Although work-related issues are major causes of mental distress among Japanese people of working age [1-3], personal concerns also have a significant effect on the mental health of this population. There are a variety of concerns in life, such as the negative consequences of marriage and personal relationships, as well as financial difficulties and illness. Relatively few studies have examined the variety of personal issues that affect psychological distress among Japanese workers, especially those which have been adjusted for work-related factors [4]. Ogami and colleagues [5], for example, found that financial matters, divorce, and illness were associated with depressive symptoms among Japanese discretionary workers who are working under the premise of the deemed working hours system. Other studies have further described how unemployment, low income, and divorce are associated with mental distress, and even with suicide, among Japanese adults, especially men [6,7].

Depression among workers represents a major occupational health concern in Japan from the viewpoint of reduced performance and substantial economic loss [8], for which various measures have been taken to minimize work stress and prevent depression among workers [9-11]. However, personal problems have not been well addressed by occupational health service because the effects of personal problems on psychological distress are not well delineated, or because healthcare workers in a clinical setting may find it difficult to address personal problems that are not health-related in a physical sense.

The Ministry of Health, Labour and Welfare (MHLW) of Japan regularly conducts comprehensive surveys of living conditions that provide a large database on individual problems and concerns, including information on a variety of personal problems, work condition issues and psychological distress [12]. The current study examined the most concerning personal problems which have the greatest impact on psychological distress among working-age Japanese men, rather than issues relating to work tasks or duties; which have often been investigated in previous research.

## Methods

### Data collection

The Ministry of Health, Labour and Welfare has conducted a comprehensive survey of living conditions in Japan every year since 1986 to help evaluate living conditions, welfare, health, and income; to assist with planning, management and policy implementation [12]. Conducted every three years, this survey covers approximately 289,363 households nation-wide in randomly selected areas. Participants are randomly selected from 5,150 municipalities of the National Census. In designated municipalities, instructors at public health centers in prefectures conduct training for persons who are in charge of the survey and conduct interviews with selected households. To conduct the present study, we requested data from the 2010 database according to procedures governing the use of official MHLW statistics. The file included de-identified data on all family members from each of the 228,664 households who had agreed to participate in the survey during 2010. We analyzed data from all men aged 20–59 years who were at the time, employed and the head of a household whose earnings maintained family.

### Questionnaire

Questions in the MHLW survey included basic demographic information, such as age, sex, occupation, and employment status. There were 13 categories of occupation: 1) management, 2) professional and technical work including teachers, health care workers and researchers (professional), 3) clerk, 4) sales, 5) service, 6) security, 7) agriculture and fishery (agriculture), 8) manufacturing, 9) transportation or machine operator (transport), 10) construction, 11) cleaning, packing operators (cleaning), 12) others, and 13) unknown. Employment status consisted of self-employed, employers of companies, regular workers, non-regular workers, and others [13].

The first question regarding the problems on present personal problems causing annoyance and stress in life was: “Do you have any problems that make you annoyed or feel stress in daily life at present (Yes/No)?” The people who answered “Yes” were asked to select all possible causes of annoyance or stress from 21 categories, and then to select the most concerning present personal problems: 1) personal relationships with family, 2) personal relationships with anyone except family, 3) love or sex, 4) marriage, 5) divorce, 6) bullying or harassment, 7) Concerned about one’s purpose in life, 8) no time for myself, 9) financial difficulties, 10) own diseases, 11) disease and care of a family member, 12) pregnancy of their wife, 13) child-rearing, 14) household chores, 15) Education for himself, 16) education of his children, 17) own tasks or duties of work, 18) family’s working conditions, 19) housing and environmental conditions, 20) others, and 21) do not know. Some respondents did not choose any items from this list even though they had answered “yes” for the first question asking if they had any problems that made them annoyed or stressed in life.

The questionnaire also included the Japanese version of the Kessler 6 (K6) which is comparable with the center for Epidemiologic Studies Depression Scale (CES-D) [14,15]. The cut-off of the K6 for determining psychological distress was over 5, based on a previously validity study of a Japanese version of the K6 with 100% sensitivity, 68.7% of specificity based on the Youden index which provided the same optimal cut-off point for the scale [15].

### Statistical analysis

We first conducted chi-square analyses to determine the associations between psychological distress and each variable of the most concerning present personal problem. Logistic regression was used to examine potential associations of the most concerning present personal problems with psychological distress, adjusting for occupational variables (occupation and employment status) and age, using problems from own tasks or duties of work as the reference [16,17]. Logistic regression calculated the odds ratios (OR) and confidence intervals (CI). All analyses were performed using IBM SPSS Statistics 20, with the level of statistical significance set at p < 0.05. All ORs were adjusted using Zhang’s correction formula for common outcomes, given that the prevalence of psychological distress was relatively high [18].

### Ethics statement

This study involved a retrospective analysis of data that had already been obtained during a national survey. As we did not use any personally identifiable information, based on regulations existing in Japan, ethical approval was not required.

## Results

The demographic characteristics of participants are shown in Table 1. The data from a total of 76,950 survey participants were analyzed in this study. The most frequent occupation was professional (28.1%) and the most frequent employment status was regular (71.7%). Nearly half (47.7%) of the sample answered that there were no annoying or stressful problems in their daily life at present, while 27.7% of the participants reporting having psychological distress. Table 2 shows the associations between the most concerning present personal problems with psychological distress. A relatively high proportion of psychological distress was associated with bullying and harassment, divorce, and a lack of purpose in life, compared with work tasks and duties.

**Table 1:** **Participant characteristics**

	**n = 76,950**	**(%)**
Age		
20-29	6,844	(8.9)
30-39	20,462	(26.6)
40-49	23,295	(30.3)
50-59	26,349	(34.2)
Occupation		
Management	9,566	(12.4)
Professional	21,590	(28.1)
Clerk	6,315	(8.2)
Sales	6,080	(7.9)
Service	7,750	(10.1)
Security	1,691	(2.2)
Agriculture	1,560	(2.0)
Manufacturing	6,955	(9.0)
Transport	3,260	(4.2)
Construction	5,713	(7.4)
Cleaning	1,992	(2.6)
Others	4,478	(5.8)
Employment status		
Self-employed	10,597	(13.7)
Employer	7,794	(10.1)
Regular	55,162	(71.7)
Non-regular	2,302	(3.0)
Others	1,095	(1.4)
The most concerning present personal problems causing annoyance and stress		
Bullying and harassment	50	(0.1)
Divorce	107	(0.1)
Concerned about one’s purpose in life	656	(0.9)
Personal relationships with family	979	(1.3)
Personal relationships with anyone except family	1,474	(1.9)
Own diseases	1,350	(1.8)
Financial difficulties	7,703	(10.0)
Love or sex	235	(0.3)
Tasks or duties of work	17,739	(23.1)
Marriage	272	(0.4)
No time for myself	998	(1.3)
Disease and care of a family member	1,408	(1.8)
Education for himself	145	(0.2)
Household chores	45	(0.1)
Family's working condition	355	(0.5)
Housing and environment	652	(0.8)
Education for his children	838	(1.1)
Child-rearing	190	(0.2)
Wife’s pregnancy	64	(0.1)
None	36,710	(47.7)
Others	1,048	(1.4)
Do not know	388	(0.5)
Not chosen	3,544	(4.6)
Psychological distress		
Distressed	21,351	(27.7)
Not distressed	55,599	(72.3)

**Table 2:** **Associations of the most concerning present personal problems with psychological distress**

	**Psychological distress**		**Not psychological distress**		**p-value**
	**n = 21,351**	**(%)**	**n = 55,599**	**(%)**	
The most concerning present personal problems causing annoyance and stress					
Bullying and harassment	36	(72.0)	14	(28.0)	<0.01
Divorce	73	(68.2)	34	(31.8)	<0.01
Concern about one’s purpose in life	423	(64.5)	233	(35.5)	<0.01
Personal relationships with family	561	(57.3)	418	(42.7)	<0.01
Personal relationships with anyone except family	808	(54.8)	666	(45.2)	<0.01
Own diseases	672	(49.8)	678	(50.2)	0.87
Financial difficulties	3,696	(48.0)	4,007	(52.0)	<0.01
Love or sex	113	(48.1)	122	(51.9)	0.56
Tasks or duties of work	7,522	(42.4)	10,217	(57.6)	<0.01
Marriage	117	(43.0)	155	(57.0)	0.02
No time for myself	421	(42.2)	577	(57.8)	<0.01
Disease and care of a family member	559	(39.7)	849	(60.3)	<0.01
Education for himself	64	(44.1)	81	(55.9)	0.16
Household chores	18	(40.0)	27	(60.0)	0.18
Family's working condition	128	(36.1)	227	(63.9)	<0.01
Housing and environment	231	(35.4)	421	(64.6)	<0.01
Education for his children	276	(32.9)	562	(67.1)	<0.01
Child-rearing	57	(30.0)	133	(70.0)	<0.01
Wife’s pregnancy	16	(25.0)	48	(75.0)	<0.01
None	3,659	(10.0)	33,051	(90.0)	<0.01
Others	391	(37.3)	657	(62.7)	<0.01
Do not know	163	(42.0)	225	(58.0)	<0.01
Not chosen	1,347	(38.0)	2,197	(62.0)	<0.01

Table 3 shows the results of multiple logistic regression analysis. This analysis revealed that psychological distress was associated with bullying and harassment (OR: 2.05, 95% CI: 1.50-2.56), divorce (OR: 1.90, 95% CI: 1.53-2.26), concern about one’s purpose in life (OR: 1.73, 95% CI: 1.59-1.88), personal relationships with family (OR: 1.49, 95% CI: 1.37-1.60), personal relationship with anyone except family (OR: 1.38, 95% CI: 1.29-1.48), own diseases (OR: 1.24, 95% CI: 1.15-1.33), and financial difficulties (OR: 1.16, 95% CI: 1.12-1.20), compared with work-related problems. We also found a negative association of psychological distress with wife’s pregnancy (OR: 0.51, 95% CI: 0.31-0.82), child-rearing (OR: 0.63, 95% CI: 0.48-0.81), children’s education (OR: 0.75, 95% CI: 0.66-0.84), housing and environment (OR: 0.79, 95% CI: 0.70-0.90), and family’s working condition (OR: 0.84, 95% CI:0.71-0.99); when compared with own work tasks or duties.

**Table 3:** **Associations between Psychological Distress and the Most Concerning Personal Problems Causing Annoyance and Stress (n = 76,950)**

	**Crude**		Multivariate*	
	**OR**	**(95% CI)**	**OR**	**(95% CI)**
The most concerning present problems causing annoyance and stress				
Bullying and harassment	2.07	(1.51-2.57)	2.05	(1.50-2.56)
Divorce	1.91	(1.54-2.26)	1.90	(1.53-2.26)
Concern about one’s purpose in life	1.75	(1.61-1.90)	1.73	(1.59-1.88)
Personal relationships with family	1.48	(1.37-1.60)	1.49	(1.37-1.60)
Personal relationships with anyone except family	1.40	(1.31-1.49)	1.38	(1.29-1.48)
Own diseases	1.23	(1.14-1.32)	1.24	(1.15-1.33)
Financial difficulties	1.17	(1.13-1.21)	1.16	(1.12-1.20)
Love or sex	1.17	(0.98-1.39)	1.12	(0.93-1.33)
Tasks or duties of work	ref		ref	
Marriage	1.02	(0.85-1.20)	0.99	(0.83-1.18)
No time for myself	0.99	(0.90-1.09)	0.98	(0.89-1.08)
Disease and care of a family member	0.92	(0.85-1.00)	0.95	(0.87-1.03)
Education for himself	1.05	(0.82-1.31)	0.99	(0.77-1.25)
Household chores	0.93	(0.58-1.40)	0.92	(0.57-1.38)
Family's working condition	0.84	(0.71-0.99)	0.84	(0.71-0.99)
Housing and environment	0.80	(0.70-0.91)	0.79	(0.70-0.90)
Education for his children	0.73	(0.69-0.97)	0.75	(0.66-0.84)
Child-rearing	0.66	(0.51-0.84)	0.63	(0.48-0.81)
Wife’s pregnancy	0.53	(0.32-0.85)	0.51	(0.31-0.82)
None	0.20	(0.19-0.21)	0.20	(0.19-0.21)
Others	0.85	(0.77-0.94)	0.87	(0.82-0.92)
Do not know	0.99	(0.85-1.14)	0.97	(0.83-1.12)
Not chosen	0.87	(0.83-0.92)	0.87	(0.82-0.92)

*Adjusted for age, occupation, and employment status.

OR: Odds ratio; CI: confidence interval; ref: referent.

## Discussion

In this study we identified several personal problems that might have a greatest impact on mental health among working-age males in Japan, perhaps more so than issues related to work tasks or duties. While such problems might be expected to be solved individually, they are usually difficult to solve by themselves. The number of individuals who were concerned about personal problems that could have a significant effect on mental health was not large; therefore, efficient screening to identify those who need support for their problems and their mental health needs to be considered.

Problems caused by interpersonal relationships are known to influence mental health outcomes, especially, their most negative consequences, such as bullying and harassment. There is ample evidence that bullying and harassment have a strong association with poor mental health outcomes [19-21]. In this study, we did not classify whether bullying and harassment occurred in the workplace or in personal relationships. Personal relationships with family and others also can be associated with psychological distress. Educational and skills-based interventions could be applicable to preventing the negative consequences of disruptive relationships [22]; however, there is insufficient evidence on the effectiveness of these interventions in working-age populations. Multidisciplinary collaboration between communities and workplaces are therefore necessary to help individuals improve their interpersonal relationships and move towards increased mental resilience.

Divorce, a generally negative outcome of marriage, is a major factor that affects the mental health of Japanese men, even increasing their risk of suicide [5,6]. Statistics on the reasons for divorce in Japan indicate that the most common reason is mismatch of characteristics (64% for male and 44% for female), followed by domestic violence, and psychological conflicts between wives and husbands [23]. These reasons might be difficult to address using interventions at the workplace, even though community-based interventions might not solve these problems either. Since the effect of divorce is so large, interventions for preventing divorce should be researched further.

Having a purpose in life, which the Japanese call *ikigai*, is a great motivation for life and has been shown to reduce the risk of mortality among elderly men [24-26]. It is often viewed as a dichotomous concept. A previous study [27], for example, reported that almost half (48.7%) of men simply answered “no” to the question “Do you have *ikigai* in your life?” However, only 0.9% of participants in the current study chose this lack of purpose in life as the problem about which they were most concerned. The exact meaning of a lack of *ikigai*, or a not having a life worth living, may differ between those of working age and the elderly. As such, further studies should now be undertaken to establish the more specific components of *ikigai*, especially the intergenerational aspects.

One’s own diseases are often recognized as risk factors for mental distress or as somatic manifestations of depression [5,28]. A variety of diseases have been suggested which may result in presenteeism and absenteeism among individuals of working age [8], including chronic pain, mental disorders, and other life-threatening diseases, such as cancer. In the current study, we did not identify what exact diseases or symptoms the participants had, although different diseases and symptoms could conceivably have significant effects on psychological distress. Therefore, further research should now be undertaken to better identify and address specific diseases and symptoms that may require the most support.

Financial difficulties were frequently listed as problems of most concern in the current study, followed by working conditions. This result is somewhat expected as financial difficulties have often been suggested as a risk factor in psychological distress during other studies [5,7,29]. In the current investigation, the presence of financial difficulties was based on each participant’s self-perception, rather than a quantitative assessment of income or indebtedness. Mortgage delinquency on housing loans is another common issue (17% of household) for the working population of Japan [30], and is known to cause anxiety about future plans in an era of economic downturn [17]. With regard to indebtedness, given that health services may find it difficult to help in this particular situation, increased collaboration between other governmental departments and service agencies might be necessary in future.

There are a few limitations of the current study. Firstly, as a cross-sectional investigation it was not possible to determine causality. Future, longitudinal research studies will therefore be needed to address these issues. Secondly, the classification of personal problems used in the original MHLW survey has not been validated in previous research. In our current study, almost half the participants did not select any items describing annoying or stressful factors in their daily life. A Survey on the State of Employees' Health conducted in Japan during 2013 for example, also reported that 40% of men responded “no” to any factors causing strong anxiety, or annoyance and stress in their work and life [31]. There may have been some participants who suffered from mental health issues or stressors issues that were not addressed in the current study. Furthermore, the validity of questions used to identify personal problems that were annoying or stressful in life is not known, and indeed, some questions in the official MHLW survey were somewhat imprecise.

## Conclusion

This study suggests that there are several personal factors which have a larger influence on the mental health status of working-age Japanese men; rather than work tasks or duties which have often been the focus of previous research. Asking workers what personal problems most concern them could help identify those in most need of psychological support. Multidisciplinary interventions for life concerns may be necessary for helping to reduce psychological distress in Japan, as elsewhere.
